# Application of Porous Nickel-Coated TiO_2_ for the Photocatalytic Degradation of Aqueous Quinoline in an Internal Airlift Loop Reactor

**DOI:** 10.3390/ijerph9020548

**Published:** 2012-02-15

**Authors:** Suiyi Zhu, Xia Yang, Wu Yang, Leilei Zhang, Jian Wang, Mingxin Huo

**Affiliations:** Department of Environmental Science and Engineering, Northeast Normal University, Changchun 130024, China; Email: zhusuiyi@163.com (S.Z.); kaimomicrobial@126.com (W.Y.); zhangll@126.com (L.Z.); wangj@126.com (J.W.)

**Keywords:** quinoline, photocatalysis, porous nickel, aeration, water purification

## Abstract

P25 film, prepared by a facile dip-coating method without any binder, was further developed in a recirculating reactor for quinoline removal from synthetic wastewater. Macroporous foam Ni, which has an open three-dimensional network structure, was utilized as a substrate to make good use of UV rays. Field emission scanning electron microscopy and X-ray diffraction analysis showed that the coated/calcinated P25 films consisted of two crystal phases, and had a number of uniform microcracks on the surface. The effects of initial quinoline concentration, light intensity, reaction temperature, aeration, and initial pH were studied. Increased reaction time, light intensity, environmental temperature, and gas aeration were found to significantly improve the quinoline removal efficiency. The aeration effect of oxygen dependency on the quinoline degradation had the trend pure oxygen > air > no gas > pure nitrogen with free O_2_. The solution pH crucially affected quinoline photodegradation; the high electrostatic adsorption of quinoline molecules on the TiO_2_ surface was strongly pH dependent. 2-Pyridine-carboxaldehyde, 3-pyridinecarboxaldehyde, and 2(1*H*)-quinolinone were identified as the major intermediates of quinoline degradation. Based on these intermediates, a primary degradation mechanism was proposed. This reusable P25 film benefits the photodegradation of water contaminants and has potential in other various applications.

## 1. Introduction

Quinoline, a representative heterocyclic nitrogen (N) compound, is widely used as a raw material in different industries (e.g., petroleum, chemical, medical, and pesticide). Due to the presence of N in this heterocyclic compound, quinoline has a low Henry constant and high water solubility. Hence, quinoline is difficultly biodegradable, tends to accumulate in natural water environments [1], and has toxic properties. Consequently, quinoline is gaining attention given the health risks it poses [2]. 

The use of TiO_2_ is an attractive technique for the complete destruction of undesirable liquid and gaseous contaminants by ultraviolet (UV) light or solar illumination. For quinoline degradation, many microscale/nanoscale catalysts like phase-pure TiO_2_, TiO_2_ (Degussa P25), doped TiO_2_, and WO_3_ [3,4,5,6] have been applied in different slurry systems. For example, the use of S-doped TiO_2_ powder in a stirred slurry reactor presented high degradation efficiency under these experimental conditions regardless of the catalyst recovery and reuse [5,6], whereas both OH·and O_2_·radicals generated in the photocatalytic process led to the opening of a heterocyclic ring [7]. Some cases mainly focused on suspended slurry systems and proved that nanometer TiO_2_ powders with high crystallinities and large specific surface areas are efficient for water purification at ambient conditions.

However, photocatalyst separation from a suspension and the aggregation of dispersed particles in a slurry reactor hamper the practical applications of TiO_2_ powders [8]. Hence, recent studies have focused on the development of nano-TiO_2_ immobilized on different substrates to increase the photocatalytic degradation efficiency as well as avoid the abovementioned separation and aggregation limitations. TiO_2_ coated/calcinated on a porous substrate has also been experimentally found to enhance the photocatalytic efficiency of TiO_2_ films by enlarging the Brunauer-Emmett-Teller (BET) surface area and TiO_2_ loaded amount [9,10]. A few researchers have reported a nano-TiO_2_-coated porous metal for the photocatalytic degradation of indoor volatile organic compounds [11]. However, to our knowledge, studies on the design and use of a photocatalytic reactor based on a TiO_2_ film-coated nickel (Ni) foam for the degradation of aqueous pollutants is very limited. Such studies have very significant implications in the treatment of industrial wastewater.

The present study aimed to investigate the applicability of a Degussa P25 TiO_2_ film coated/calcinated on porous Ni in a new tubular photocatalytic reactor for the minimization of model wastewater containing quinoline. The influence of various parameters (*i.e.*, initial concentration, environmental temperature, light intensity, aeration, and pH) on the efficiency of the process was assessed to optimize the degradation. The photocatalytic stability of the P25 film was also evaluated.

## 2. Materials and Methods

### 2.1. Materials

Quinoline (C_9_H_7_N, MW = 129) was acquired from Sinopharm, China, and used without further purification. TiO_2_ (P25; ca. 80% anatase and 20% rutile) was purchased from Deguessa, Germany, and had a BET surface area of 55 m^2^/g. Anhydrous alcohol was obtained from Sigma. The natural pH of the synthetic wastewater containing quinoline was 6.7. Prior to photodegradation, the pH of the wastewater was adjusted using a small amount of either H_2_SO_4_ or NaOH, and was monitored by a digital pH meter (WTW Multi 340i, Germany). All chemicals used were reagent grade. The distilled water used to prepare all sample solutions was prepared in our laboratory.

### 2.2. Preparation and Characterization of the Photocatalyst

Degussa P25 TiO_2_ was immobilized on porous Ni (denoted as TiO_2_/Ni) via a facile dip-coating method without the use of coating equipment, as reported in our previous study [12]. P25 TiO_2_ was added to anhydrous alcohol [13], and then dispersed for 10 min under ultrasonic agitation to confect the P25 sol to 10% concentration. 

Foam Ni with a specific surface area of 0.25 m^2^/g was used as a support because of its homogeneous vesicular structure and facile machining properties, which are beneficial to increasing the contact of photocatalysts with UV rays [14,15]. Pre-dried foam Ni was cut into tubes of 40 cm × Ø 7 cm, and then immersed into the P25 sol for 20 s. The tubes were taken out and air dried at room temperature. After ethanol volatilization overnight, the TiO_2_/Ni film was calcined at 90 °C for 60 min, and then at 450 °C for 30 min at a heating rate of 3 °C/min in a muffle furnace. The TiO_2_/Ni film obtained was washed with boiling water to remove uncoated TiO_2_ particles. The surface morphology of the prepared TiO_2_/Ni film and bare porous Ni substrate were observed using a field emission scanning electron microscopy (FESEM) system (XL-30 ESEM FEG, Philips). The crystal phase structures were measured by X-ray diffraction (XRD) on a Rigaku D/max-3c diffractometer (CuKα radiation, *λ* = 0.15405 nm). The specific surface area of the prepared TiO_2_/Ni film was 8.5 m^2^/g, as determined by nitrogen adsorption-desorption measurements (TriStar 3000). 

### 2.3. Photocatalytic Reactor

**Figure 1:**
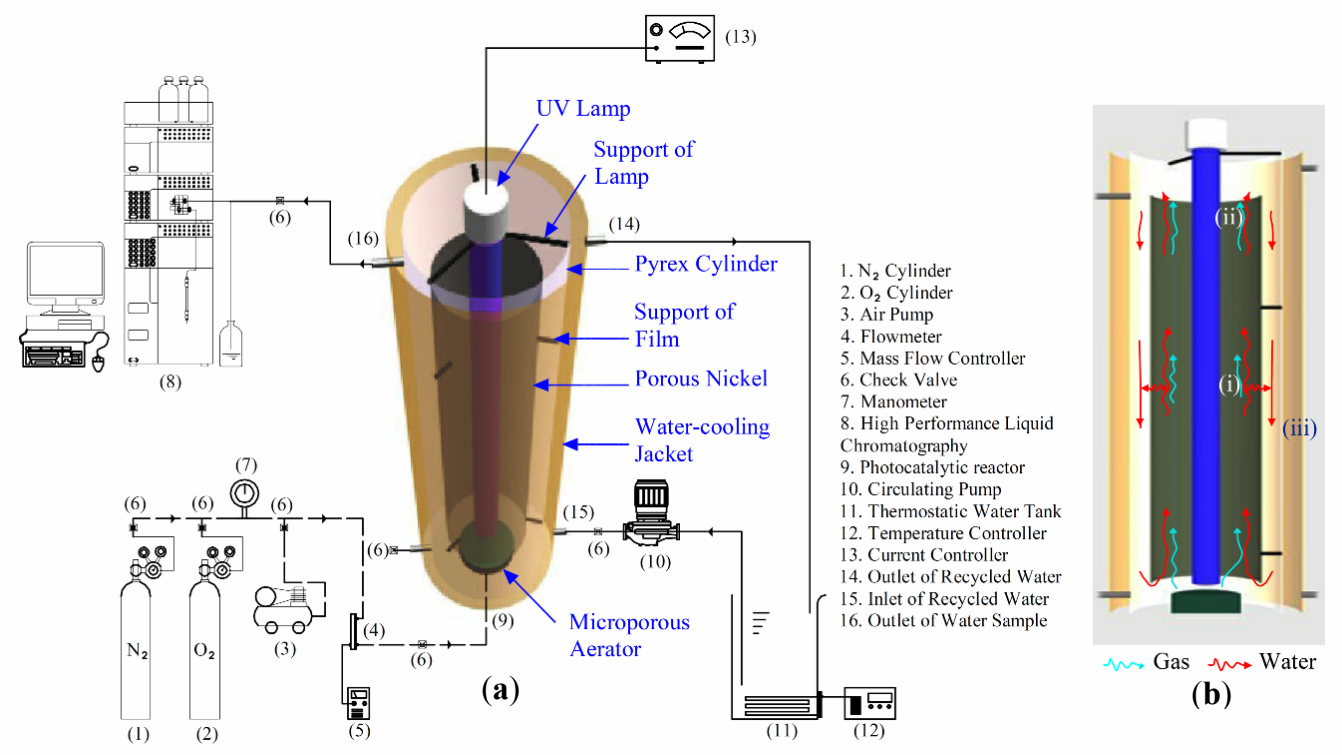
Schematic diagram (**a**) and cross section (**b**) of the tubular photocatalytic reactor.

An internal airlift loop photocatalytic reactor made of Pyrex glass was used for the photocatalytic experiments, as shown in Figure 1. The effective volume of the reactor was 3.5 L, and the dimensions were 55 cm × Ø 10 cm. A UV lamp (15 W, 254 nm) was longitudinally fixed in the center of the reactor. The prepared tubular TiO_2_/Ni film was placed on a fixed support outside the UV lamp, resulting in a 2.5 cm gap. A microporous aerator was fixed below the reactor to maintain aerobic conditions inside the TiO_2_/Ni tube. Gas bubbled up from the bottom and accelerated around the liquid. The liquid inside the TiO_2_/Ni tube flowed in two directions: (i) some liquid gradually flowed out through the tube pores, and (ii) the others flowed up to the top of the tube. Consequently, the liquid outside the tube flowed down from the top; (iii) due to the different internal and external flow rates, resulting in water circulation in the photocatalytic reactor. This process benefited the enhancement of the contacting probability between the water contaminant and the P25 TiO_2_ on the surface of porous Ni. The experimental temperature of the entire photoreactor was controlled by a surrounding external water-cooling jacket.

### 2.4. Procedure and Analysis

In the photocatalytic process, 3.5 L of aqueous quinoline solution was poured into the reactor. The UV lamp was switched on to start the photo-oxidation process. Compressed gas was bubbled upward through the gas distributor at the bottom of the reactor. At pre-defined irradiation times, 50 μL of solution was collected and immediately analyzed to avoid further reaction. The dependences of the quinoline degradation rate on initial concentration, light intensity, reaction temperature, aeration, and pH were investigated. Each experiment was conducted three times under identical conditions. The data presented in the text and figures are the mean values. The light absorption characteristics of the solutions were recorded using Persee T6 UV/visible spectrophotometer at a distance of 100 cm from the light source per the manufacturer’s instructions [16]. The concentration of quinoline was analyzed using a high-performance liquid chromatography system (LC-20A). The chromatographic column was a Shim-pack VP-ODS-C18 (4.6 mm × 150 mm, 5 μm). The mobile phase was a mixture of methanol and water (60:40, v/v) with a flow rate of 1 mL/min. The testing wavelength was 275 nm. 

To obtain the intermediate products of quinoline degradation, the experiment was performed in a photocatalytic reactor with 100 mg/L quinoline under ambient conditions. After 30 min of irradiation time, 200 mL of the effluent was collected in a separating funnel, and then extracted for qualitative analysis by gas chromatography (GC)/mass spectroscopy (MS). The effluent for the GC/MS sample was further assayed by the method of Li *et al*. [17], except that 30 mL of anhydrous ether was used as the extractant. The extracts were decanted into a rotary evaporator, and then dried at 35 °C to evaporate the ether. The residue was dissolved in 10 mL of anhydrous ether for the GC/MS analysis. 

GC/MS analysis was performed on an Agilent GC system (6890N/MSD 5973) equipped with an HP-5MS fused silica capillary column (30 m × 0.25 mm i.d., 0.25 μm film thickness). The column was first heated to 80 °C, kept constant for 3 min, linearly programmed to increase to a final temperature of 300 °C at a rate of 10 °C/min, and then isothermally held for 2 min. The temperature for the MS ion source was 200 °C, and the electron energy was 70 eV. 

## 3. Results and Discussion

### 3.1. TiO_2_/Ni Film Characterizations

**Figure 2:**
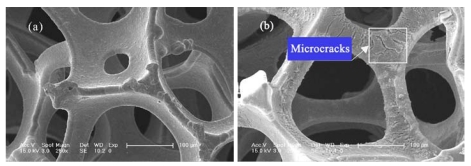
FESEM images of porous nickel substrate (**a**) and the TiO_2_/Ni film (**b**).

The FESEM images of porous Ni and the TiO_2_/Ni film are shown in Figure 2. The surface of bare porous Ni without immobilized P25 was smooth (Figure 2a). After P25 TiO_2_ coating, the originally smooth surface of the coated TiO_2_/Ni film (Figure 2b) became relatively rough and had a number of microcracks. Then, the specific surface area increased remarkably from 0.25 m^2^/g to 8.5 m^2^/g. Consequently, a high photocatalytic efficiency was obtained because there were more interfaces between the catalyst and reactants. 

**Figure 3:**
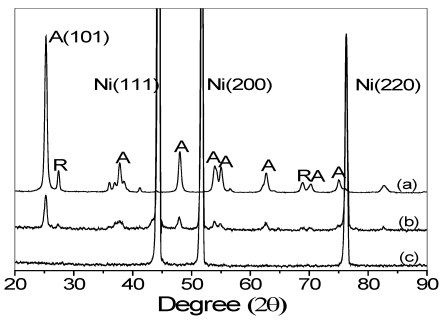
XRD pattern of Degussa P25 TiO_2_ (**a**), the TiO_2_/Ni film (**b**) and the bare nickel substrate (**c**). A and R denotes the anatase and rutile crystal phases, respectively.

Figure 3 shows the XRD patterns of P25 TiO_2_ powder, TiO_2_/Ni film, and porous Ni. Three strong diffraction peaks found at 2*θ* = 44.5°, 51.8°, and 76.1° were ascribed to the (111), (200), and (220) planes of Ni (ICDD PDF Card 87-0712) (JCPDS. 2001), respectively, in accordance with earlier studies [18,19]. 

Two diffraction peaks at 2*θ* = 37.2° and 68.3° were attributed to rutile phase (ICDD PDF Card 21-1276) [20]. The presence of other crystallographic plains labeled with “A” belongs to the anatase phase (ICDD PDF Card 21-1272) [20]. P25 TiO_2_ coated on porous Ni caused no apparent change in the half width of the anatase (101) XRD peak, regardless of the heating treatment, and the crystallite size was calculated to be 21 nm from the anatase (101) peak width using the Scherrer equation. This result indicated that nano-sized TiO_2_ particles in P25 sol have been reproduced onto the surface of porous Ni. Therefore, the TiO_2_/Ni film possessed good photocatalytic activity.

### 3.2. Photodegradation of Quinoline in the Photocatalytic Reactor

**Figure 4:**
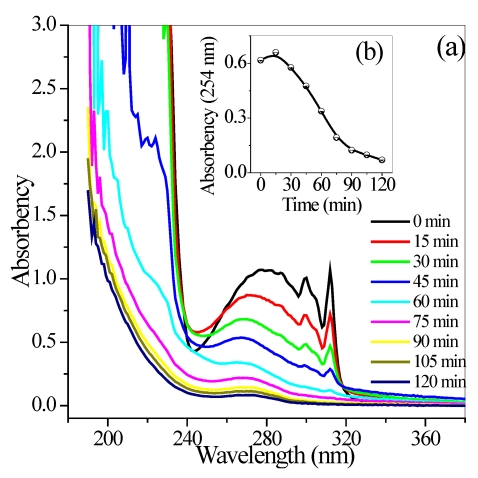
Absorbance curves (**a**) and UV_254_ (**b**) of aqueous quinoline solution. Conditions: *C*_0_ = 40 mg/L, *T* = 290 K, flow rate = 0.35 L/min, pH = 6.7, and the maximum irradiation intensity.

To analyze the degradation process of quinoline, a UV absorption experiment was performed on the reactants, as described in the Materials and Methods Section. With increased illumination time, a series of UV absorption spectra was recorded within the 180–380 nm range, as shown in Figure 4a. The characteristic absorption of quinoline significantly decreased with increased irradiation time, and almost disappeared at 60 min. The UV_254_ of the reaction solution initially increased and then gradually decreased. The peak appeared at 15 min (Figure 4b). This finding indicated that the aromatic or pyridine ring of quinoline opened in the initial stage, and formed a series of intermediate products containing conjugated double bond. As a result, UV_254_ absorbance was enhanced. UV absorbance within the range 180–240 nm also gradually decreased with prolonged irradiation time. This result indicated the further mineralization of aliphatic saturated as well as cyclic hydrocarbons and their derivatives in solution [21]. Interestingly, UV absorbance above 320 nm initially increased, reached the peak at 45 min, and then apparently decreased. These findings were in accordance with the color of the reaction solution changing from transparent to pale yellow, to yellow-green, and then back to transparent.

#### 3.2.1. Effect of the Initial Quinoline Concentration

**Figure 5:**
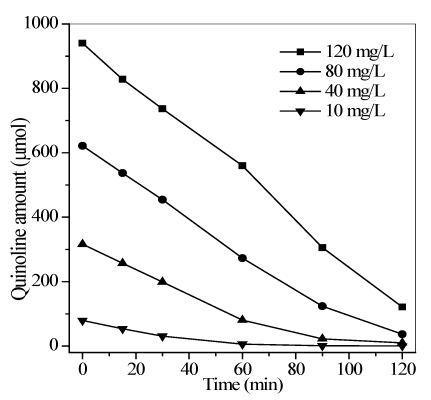
Effect of the initial quinoline concentration on the photocatalytic degradation. Conditions: *T* = 290 K, flow rate = 0.35 L/min, pH = 6.7, and the maximum irradiation intensity.

The effect of the initial quinoline concentration on the degradation efficiency was investigated within the range 10–120 mg/L, as shown in Figure 5. An initial aqueous quinoline concentration of 10 mg/L can almost be completely removed after 60 min of irradiation. Increased quinoline concentrations from 10 mg/L to 120 mg/L resulted in the increase in irradiation time. Moreover, the total removal amount of quinoline also increased from 78.74 μmol to 818.78 μmol. This result suggested that increased initial concentration obviously decreased the degradation ratio, but resulted in a high total removal amount of quinoline. Generally, the formation of activated radicals (e.g., OH and O_2_^−^) on the TiO_2_ surface remains constant for a given light intensity, catalyst amount, air flow, and duration of irradiation. A higher quinoline concentration corresponded to a higher demand for activated radicals, resulting in prolonged reaction time for the complete decomposition. However, high initial concentrations may prompt the contact between the active radicals and quinoline molecules, thereby improving the total amount of quinoline removed. 

#### 3.2.2. Effect of Light Intensity

**Figure 6:**
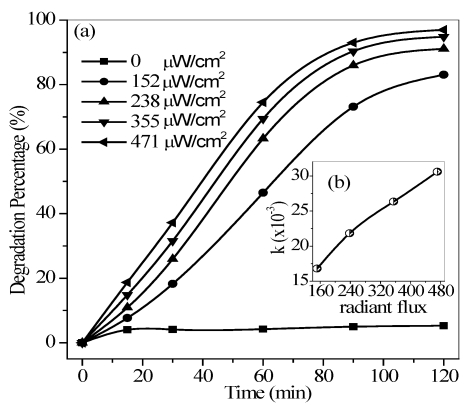
Degradation percentage (**a**) and reaction rate (**b**) with respect to different radiant flux. Conditions: *T* = 290 K, flow rate = 0.35 L/min, pH = 6.7, and *C*_0_ = 40 mg/L.

A control signal processed by a digital PID regulator was used to control the input current and change the UV light output intensity. Figure 6a shows that under dark conditions, only about 5% of the quinoline was removed from synthetic water during the first 15 min, and remained constant for nearly 100 min regardless of air flow. This finding suggested that the main removal effect in the absence of UV irradiation is due to the adsorption on the TiO_2_ surface [22]. Interestingly, the oxidation and air stripping controlled by the gas aeration had little effect on quinoline photodegradation. However, UV irradiation benefited the removal efficiency of quinoline in water, and the degradation percent reached over 80% after 120 min of reaction at 152 μw/cm^2^ of light intensity. Increased light intensity also resulted in increased photodegradation efficiency. This finding may be due to the fact that the rate of electron-hole pair formation at the TiO_2_ surface increased with increased light intensity to improve the photocatalytic efficiency. Figure 6b shows an acceptably good linear correlation between the apparent first-order rate constant and light intensity. This tendency indicated that light intensity is an important control agent of photocatalytic reaction within the radiant flux range of 152 to 471 μW/cm^2^. 

#### 3.2.3. Effect of Reaction Temperature

**Figure 7:**
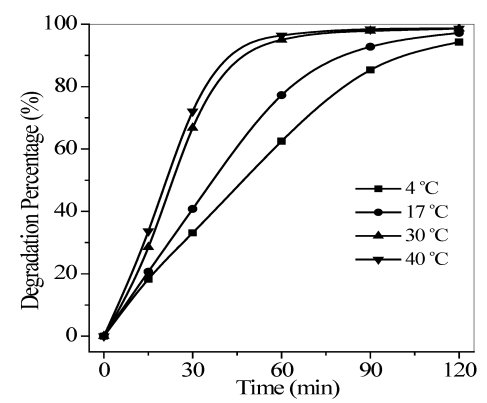
Effect of temperature on the photocatalytic degradation of quinoline. Conditions: flow rate = 0.35 L/min, pH = 6.7, *C*_0_ = 40 mg/L, and the maximum irradiation intensity.

The dependence of the quinoline degradation rate on the reaction temperature within 4–40 °C was also determined. Figure 7 shows that compared with the degradation percent at 60 min, the degradation ratio rapidly increased within 4–30 °C, and then very gradually increased above 30 °C. 

Warm temperatures enhance the collision frequency of molecules [23]. Hence, the movement of the reactant molecules continued to intensify and gradually reached a plateau. However, for TiO_2_ photocatalysis, electron-hole pair generation at ambient temperatures is mainly affected by light irradiation given that the band gap energy is too high to be overcome by thermal activation [24]. Therefore, the increase in percent quinoline degradation was most likely due to the enhanced collision frequency with the increased reaction temperature.

#### 3.2.4. Effect of Aeration

**Figure 8:**
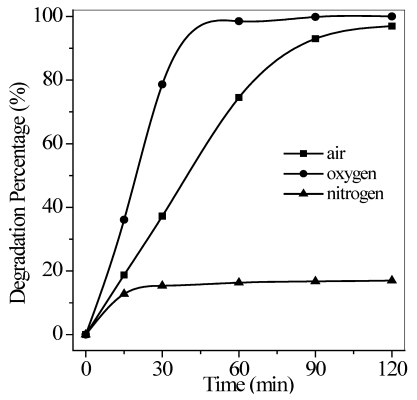
Effect of air, pure nitrogen, and pure oxygen aeration on the photodegradation of quinoline. Conditions: flow rate = 0.35 L/min, pH = 6.7, *T* = 17 °C, *C*_0_ = 40 mg/L, and the maximum irradiation intensity.

Molecular oxygen is generally used as an electron acceptor to capture photo-generated electrons and form an active radical (O_2_^−^). Therefore, quantum efficiencies may partly be increased by inhibiting electron-hole recombination to enhance the photocatalytic efficiency [25]. In the present study, compressed pure oxygen, pure nitrogen, and air were purged into the photocatalytic reactor to analyze the effect of dissolved oxygen on quinoline photodegradation. Figure 8 shows that the degradation percent of quinoline solution under pure oxygen aeration at the rate of 0.35 L/min reached 97% after continuous UV illumination for 60 min. This percentage was much higher than that under airflow or pure nitrogen aeration. Under pure nitrogen aeration, the percent quinoline degradation rapidly increased for the first 15 min, and then became constant with time. Therefore, molecular nitrogen clearly existed in air, and pure nitrogen aeration played a negative role in the photocatalytic reaction, playing a dual role. One was to reduce residual dissolved oxygen in water by continued stripping. The other was to relocate dissolved oxygen onto the TiO_2_ surface to inhibit the formation of active radicals, and retard the agitation effect to decrease the effective mass transfer for the photocatalytic process. 

**Figure 9:**
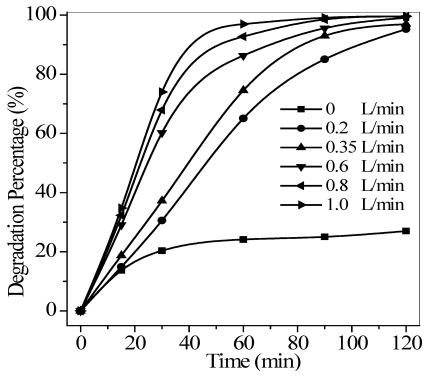
Effect of air flow on the photocatalytic degradation of quinoline. Conditions: pH = 6.7, *T* = 17 °C, *C*_0_ = 40 mg/L, and the maximum irradiation intensity.

The relationship between airflow and degradation efficiency was also investigated, as shown in Figure 9. Percent quinoline degradation increased with increased air flow rate to 1.0 L/min, which suggested that higher air aeration rate favors to increase dissolving oxygen in water and agitate the solution to speed up mass transfer.

However, compared with pure nitrogen aeration, percent quinoline degradation under no airflow was still up to 20% after irradiation for 120 min, a little above 5% of that under pure nitrogen aeration at a rate of 0.35 L/min, as illustrated in Figure 8, although a similar increased removal tendency presented in the initial stage because of the effect of residual dissolved oxygen. It’s proposed that molecular oxygen was recycled in a single photocatalytic system such that reoxygenation equilibrium existed between oxygen consumption and steady-state replenishment. The reoxygenation processes may have also proceeded at low levels under no airflow, but were inhibited by pure nitrogen stripping. 

#### 3.2.5. Effect of pH

**Figure 10:**
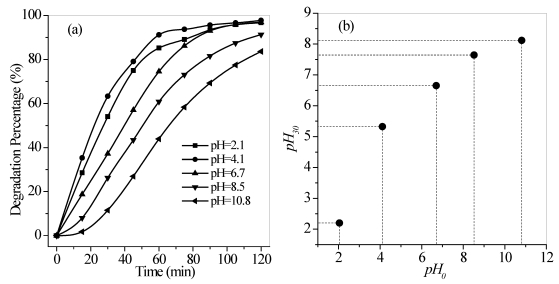
Effect of pH (**a**) on the photocatalytic degradation of quinoline and (**b**) after irradiation for 30 min. Conditions: flow rate = 0.35 L/min, *T* = 17 °C, *C*_0_ = 40 mg/L, and the maximum irradiation intensity. pH_0_ and pH_30_ represent the pH values of the aqueous solution after UV irradiation for 0 and 30 min, respectively.

The pH value of a solution determines the surface charge of photocatalyts and the ionization or speciation (pK_a_) of an organic pollutant [26,27]. Hence, pH varies significantly and can play an important role in the photocatalytic degradation of organic contaminants [28]. The effect of pH on quinoline degradation was investigated, as shown in Figure 10a. The degradation rate significantly increased at pH below 4.1, and then gradually decreased with increased pH from 6.7 to 10.8. An alkaline medium appeared to retard quinoline degradation, with the degradation percent below 10% during the first 15 min. A combined pH change analysis Figure 10b) revealed that after UV irradiation for 30 min, the initial pH value of the solution shifted to the value of the point of zero charge (*P*_zc_) of TiO_2_ (Deguessa P25), which is widely reported at pH 6.25 [29], *i.e.*, the initial pH increased from 4.1 to 5.3. These observed behaviors can be explained in terms of the *P*_zc_ of TiO_2_ and the pK_a_ (about pH 4.95) of quinoline [30]. At more alkaline pH values, the TiO_2_ surface is negatively charged. At pH values blow 6.25, the surface is positively charged [31].

When the pH is above 6.25, the adsorption of quinoline molecules onto the TiO_2_ surface became difficult with increased pH due to the repellant effect of the two species with negative charges. However, within pH 4.95–6.25, the negatively charged quinoline molecules were easily adsorbed on the positively charged TiO_2_ surface. This phenomenon benefited the quinoline degradation. At pH values below the quinoline pKa value, the quinoline molecules are present in the protonated form (with a positive charge), which did not favor the absorption onto the TiO_2_ surface, which also had a positive charge. The protonated form appeared to enhance the UV absorbance of quinoline, which led to the improvement in its degradation under acidic conditions compared with alkaline medium [32]. Also, at low pH values, a number of sulfur anions were found when the pH of the water was adjusted. These anions strongly hindered the generation of active radicals via competitive adsorption with O_2_, H_2_O, and other molecules on the catalyst surface, resulting in decreased quinoline degradation in acidic medium. Therefore, the different pH values before and after the reaction for 30 min revealed that quinoline had a higher degradation rate at pH 4.95–6.25, whereas the degradation rate was moderately influenced by the solution pH. 

#### 3.2.6. Stability of the TiO_2_/Ni Film

**Figure 11:**
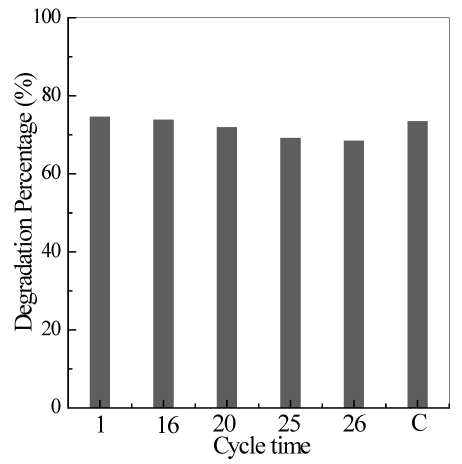
Percent quinoline degradation of the TiO_2_/Ni film with consecutive runs under the conditions of *T* = 290 K, flow rate = 0.35L/min, pH = 6.7, *C*_0_ = 40mg/L, and the maximum irradiation flux. C denotes the re-calcinated TiO_2_/Ni film.

The stability of the TiO_2_/Ni film in the fabricated reactor is one of the most important factors for its practical application. Therefore, a recycling experiment was carried out using the same TiO_2_/Ni film to investigate its stability. After each experiment, the TiO_2_/Ni film was washed with distilled water for 30 min under air aeration, dried at 105 °C for 60 min in an electric oven, and then reused under oneset of reaction conditions ([quinoline]_0_ = 40 mg/L, pH = 6.7, flow rate = 0.35 L/min, reaction temperature = 17 °C, and the maximum irradiation flux). An irradiation time of 60 min was selected for the recycling experiment due to the disappearance of the characteristic absorption of quinoline, as described in Figure 4. Figure 11 shows that the percent quinoline degradation remained constant during the first 16 runs of photocatalytic reaction, obviously decreased in subsequent runs, and then declined to 70% after 25 runs. This finding was in accordance with the TiO_2_/Ni film varying in color from white to pale yellow. However, complete reactivation occurred after the TiO_2_/Ni film was subjected to calcinations. This reactivation was due to the decomposition of the adsorbed reactants at a high temperature.

### 3.3. Mechanism of Quinoline Degradation

**Figure 12:**
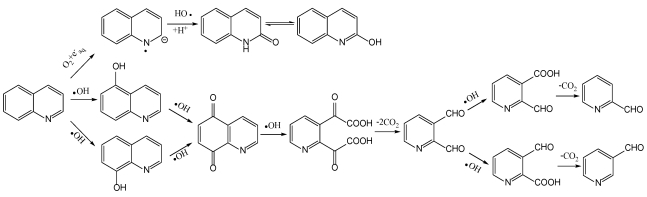
Proposed pathways for the photocatalytic degradation of quinoline.

The synthetic wastewater containing quinoline varied in color, as described in Section 3.2, indicating that there existed some intermediate products in the photocatalytic process. Based on the GC/MS analysis performed, the effluent of the synthetic wastewater after irradiation for 30 min contained 2-pyridinecarboxaldehyde, 3-pyridinecarboxaldehyde, quinoline, and 2(1*H*)-quinolinone. Figure 12 shows the possible mechanisms for the formation of the three intermediate products. The formation of the first two intermediates may have depended on the electrophilic substitution between quinoline and hydroxyl radicals. The initial oxidation mainly occurred at positions 5 and 8 of the aromatic ring, resulting in the formation of 5,8-quinolinedione. Further oxidation by active radicals resulted in the formation of 2-pyridinecarboxaldehyde or 3-pyridinecarboxaldehyde based on the phenyl ring opening process via quinoline-5,8-dione. This mechanism was in accordance with that reported by Thomsen [33] and Zhu *et al*. [34]. Considering that the electron cloud density of the pyridine ring is lower than that of an aromatic ring, the pyridine ring is relatively difficult to be oxidized. Hence, the formation of 2(1*H*)-quinolinone is related to the superoxide ion (O_2_^−^) generated by dissolved oxygen on the TiO_2_ surface trapped electron under UV irradiation. Ishibashi *et al*. [35] have reported that the superoxide ion (O_2_^−^) is generated at the water-TiO_2_ interface with a longer lifetime of about 70 s. Its absolute number pre-illuminated is calculated to be 2 × 10^14^/cm^2^, and it has a low mobility [36]. During the adsorption of quinoline onto the TiO_2_ surface, the initial addition reaction between quinoline and the superoxide ion (O_2_^−^) may take place at the C-2 position of the pyridine ring, similar with an ordinary nucleophile [34,37]. This phenomenon benefited the further reaction with active radicals to generate 2(^1^*H*)-quinolinone. 

## 4. Conclusions

A newly developed tubular photocatalytic reactor equipped with a TiO_2_/Ni film was used for the photodegradation of an aqueous quinoline solution under different operational conditions. The results showed that percent quinoline removal increased with increased light intensity and air aeration. A lower photocatalytic degradation rate was shown under pure nitrogen aeration than under pure oxygen, air, or without any aeration. Percent quinoline removal decreased with increased total quinoline amount removal from 10 to 104.4 mg/L when the initial quinoline concentration was increased from 10 to 120 mg/L. The reaction temperature and pH should be at 30 °C and below 6.25, respectively, to obtain a high removal efficiency of quinoline from synthetic wastewater. The major intermediates of quinoline degradation were identified as 2-pyridinecarboxaldehyde, 3-pyridinecarboxaldehyde, and 2(1*H*)-quinolinone. A primary degradation mechanism was proposed based on these intermediates. The new internal airlift loop tubular photocatalytic reactor based on a TiO_2_/Ni film was proven to be very effective for quinoline removal from synthetic wastewater. The use of this reactor could hopefully be extended to treat other types of industrial wastewater.
